# Supplementary material for: From sequence to enzyme mechanism using multi-label machine learning
Source: BMC Bioinformatics. 2014 May 19;15:150. doi: 10.1186/1471-2105-15-150 (PMC4229970; doi:10.1186/1471-2105-15-150)
Supplement: Additional file 2 — Java code of ml2db. Additional file ml2db_code.tar.gz contains the Java source code to run the multi-label machine learning experiments and save the results to database. The code’s Javadoc is included. [file 1471-2105-15-150-S2.zip › additional file 2/ml2db/ml2db/doc/index-files/index-9.html]

I-Index


JavaScript is disabled on your browser.


- Overview
- Package
- Class
- Use
- Tree
- Deprecated
- Index
- Help

- Prev Letter
- Next Letter

- Frames
- No Frames

- All Classes

A B C D E F G H I J K L M N O P Q R S T U V W X 


## I

IdentifiedSparseInstance - Class in main.ml2db.weka
:   Class

IdentifiedSparseInstance() - Constructor for class main.ml2db.weka.IdentifiedSparseInstance


identifier() - Method in class main.ml2db.mulan.mulanarff.IdInstance


IdInstance - Class in main.ml2db.mulan.mulanarff
:   A Weka instance with identifier.

IdInstance(String, Instance) - Constructor for class main.ml2db.mulan.mulanarff.IdInstance


IdInstanceTest - Class in test.ml2db.mulan.mulanarff
:   Class

IdInstanceTest() - Constructor for class test.ml2db.mulan.mulanarff.IdInstanceTest


IdMultiLabelInstances - Class in main.ml2db.mulan.mulanarff
:   A set of identified Mulan Multi Label instances (each instance has a unique
    string identifier).

IdMultiLabelInstances(int, DbManagers) - Constructor for class main.ml2db.mulan.mulanarff.IdMultiLabelInstances


IdMultiLabelInstancesTest - Class in test.ml2db.mulan.mulanarff
:   Class

IdMultiLabelInstancesTest() - Constructor for class test.ml2db.mulan.mulanarff.IdMultiLabelInstancesTest


ImportArff - Class in main.ml2db.weka
:   Class to import an existing Weka ARFF file to a record in the arff database table.

ImportArff(String, String, String) - Constructor for class main.ml2db.weka.ImportArff


ImportMulanArff - Class in main.ml2db.mulan.mulanarff


ImportMulanArff(String, String, String, String) - Constructor for class main.ml2db.mulan.mulanarff.ImportMulanArff


initialisationIsCorrect() - Method in class main.ml2db.weka.DataSetManager


initialise(ExperimentDetails, int, String, int) - Method in class main.ml2db.al.ActiveLearning


initialise() - Method in class main.ml2db.al.utility.confidence.ConfidenceMetric


initialise() - Method in class main.ml2db.al.utility.globaldistance.GlobalDistanceMetric


initialise(IdMultiLabelInstances, IdMultiLabelInstances) - Method in class main.ml2db.al.utility.globaldistance.GlobalDistanceMetric


initialise() - Method in class main.ml2db.al.utility.neighbours.NeighbourhoodNetwork


initialise() - Method in class main.ml2db.al.utility.pickers.byfrequency.ByFrequencyPicker


initialise(IdMultiLabelInstances) - Method in class main.ml2db.al.utility.pickers.byfrequency.NearestNeighboursByFreqUtility


initialise() - Method in class main.ml2db.al.utility.pickers.LabelRandomPicker


initialise() - Method in class main.ml2db.al.utility.pickers.RandomAttributePicker


initialise() - Method in class main.ml2db.al.utility.pickers.RandomAttributeSetPicker


initialise() - Method in class main.ml2db.al.utility.pickers.RandomInstancePicker


initialise() - Method in class main.ml2db.al.utility.pickers.RandomLabelsPicker


initialise() - Method in class main.ml2db.al.utility.pickers.RandomNeigbourhoodPicker


initialise() - Method in class main.ml2db.al.utility.UtilityMetric


initialise(String) - Method in class test.ml2db.dataharness.DataTableTest


InputAndOutputDbManagersTest - Class in test.ml2db
:   Class

InputAndOutputDbManagersTest() - Constructor for class test.ml2db.InputAndOutputDbManagersTest


INST1 - Static variable in class test.ml2db.dataharness.DataOne


INST1 - Static variable in class test.ml2db.dataharness.DataTwo


INST2 - Static variable in class test.ml2db.dataharness.DataOne


INST2 - Static variable in class test.ml2db.dataharness.DataTwo


INST3 - Static variable in class test.ml2db.dataharness.DataOne


INST3 - Static variable in class test.ml2db.dataharness.DataTwo


INST4 - Static variable in class test.ml2db.dataharness.DataOne


INST4 - Static variable in class test.ml2db.dataharness.DataTwo


INSTANCE - Static variable in class main.ml2db.al.database.AlDbCreator
:   the instance unique name

INSTANCE - Static variable in class main.ml2db.mulan.predict.PredictionsRecordTable


INSTANCE\_1\_2 - Static variable in class test.ml2db.dataharness.TestProjectParameters


INSTANCE\_1\_2\_EMPTY - Static variable in class test.ml2db.dataharness.TestProjectParameters


INSTANCE\_3 - Static variable in class test.ml2db.dataharness.TestProjectParameters


INSTANCE\_ATTRIBUTE\_QUERY - Static variable in class test.ml2db.dataharness.ArffPropsQueriesOneTest


INSTANCE\_ATTRIBUTE\_QUERY - Static variable in class test.ml2db.dataharness.ArffPropsQueriesTwoTest


INSTANCE\_ATTRIBUTE\_QUERY\_PROP - Static variable in class main.ml2db.weka.ArffPropsFromFile
:   The query to get the attributes for each instance

INSTANCE\_CLASS\_QUERY - Static variable in class test.ml2db.dataharness.ArffPropsQueriesOneTest


INSTANCE\_CLASS\_QUERY - Static variable in class test.ml2db.dataharness.ArffPropsQueriesTwoTest


INSTANCE\_CLASS\_QUERY\_PROP - Static variable in class main.ml2db.weka.ArffPropsFromFile
:   The query to get the class value for each instance

INSTANCE\_FIELD - Static variable in class test.ml2db.dataharness.DataTableTest


INSTANCE\_STRING - Static variable in class main.ml2db.al.database.QueryTable


instances1 - Static variable in class test.ml2db.mulan.measuremetrics.AnnotationsReaderTest


instancesColumn() - Static method in class test.ml2db.dataharness.DataOne


instancesFactory() - Static method in class test.ml2db.mulan.mulanarff.MulanInstancesFillerTest


instancesFactory() - Static method in class test.ml2db.weka.InstancesFillerTest


InstancesFiller - Class in main.ml2db.weka
:   Generates and fills individual instances for a weka data set.

InstancesFiller(DataSetGenerator) - Constructor for class main.ml2db.weka.InstancesFiller


InstancesFillerTest - Class in test.ml2db.weka
:   Class

InstancesFillerTest() - Constructor for class test.ml2db.weka.InstancesFillerTest


instancesValues() - Static method in class test.ml2db.dataharness.DataOne
:   Results of a select distinct instance, class query

InstanceUtils - Class in main.ml2db.mulan.mulanarff
:   Class

InstanceUtils() - Constructor for class main.ml2db.mulan.mulanarff.InstanceUtils


InstAttValueMap - Class in main.ml2db.weka
:   Contains [instance identifier, attribute name, attribute value] tuples.

InstAttValueMap(boolean, String) - Constructor for class main.ml2db.weka.InstAttValueMap


InstAttValueMap(boolean, Vector<String>, Vector<String>, Vector<String>, String) - Constructor for class main.ml2db.weka.InstAttValueMap


InstAttValueMapTest - Class in test.ml2db.weka


InstAttValueMapTest() - Constructor for class test.ml2db.weka.InstAttValueMapTest


INSTATTVOID - Static variable in class test.ml2db.dataharness.DataOne


INSTCLASSATTVOID - Static variable in class test.ml2db.dataharness.DataOne


INSTCLASSVOID - Static variable in class test.ml2db.dataharness.DataOne


internallyCrossValidate(int, int, IdMultiLabelInstances, MultiLabelLearner, boolean, boolean, boolean) - Method in class main.ml2db.mulan.learn.crosseval.CrossEvaluator
:   Evaluates a `MultiLabelLearner` via cross-validation on given data
    set with defined number of folds.

intialise() - Method in class main.ml2db.weka.AttributeFactory


isDebug() - Method in class main.ml2db.al.utility.pickers.RandomPicker


isEmptyLabelledSet() - Method in class main.ml2db.al.utility.globaldistance.GlobalDistanceMetric


isGenerateGraphViz() - Method in class main.ml2db.al.ActiveLearning


isLabelled() - Method in class main.ml2db.al.utility.neighbours.Neighbourhood


isMassiveFile() - Method in class main.ml2db.mulan.mulanarff.MulanArff


isStrict() - Method in class main.ml2db.mulan.learn.evaluation.EvaluatorNoAUC


isTest() - Method in class main.ml2db.al.ActiveLearning


isTest() - Method in class main.ml2db.al.utility.pickers.RandomPicker


isWriteToFile() - Method in class main.ml2db.al.utility.globaldistance.DistancesCalculator

A B C D E F G H I J K L M N O P Q R S T U V W X

- Overview
- Package
- Class
- Use
- Tree
- Deprecated
- Index
- Help

- Prev Letter
- Next Letter

- Frames
- No Frames

- All Classes
